# ASPDock: protein-protein docking algorithm using atomic solvation parameters model

**DOI:** 10.1186/1471-2105-12-36

**Published:** 2011-01-27

**Authors:** Lin Li, Dachuan Guo, Yangyu Huang, Shiyong Liu, Yi Xiao

**Affiliations:** 1 Biomolecular Physics and Modelling Group, Department of Physics, Huazhong University of Science and Technology, Wuhan 430074, Hubei, PR China

## Abstract

**Background:**

Atomic Solvation Parameters (ASP) model has been proven to be a very successful method of calculating the binding free energy of protein complexes. This suggests that incorporating it into docking algorithms should improve the accuracy of prediction. In this paper we propose an FFT-based algorithm to calculate ASP scores of protein complexes and develop an ASP-based protein-protein docking method (ASPDock).

**Results:**

The ASPDock is first tested on the 21 complexes whose binding free energies have been determined experimentally. The results show that the calculated ASP scores have stronger correlation (r ≈ 0.69) with the binding free energies than the pure shape complementarity scores (r ≈ 0.48). The ASPDock is further tested on a large dataset, the benchmark 3.0, which contain 124 complexes and also shows better performance than pure shape complementarity method in docking prediction. Comparisons with other state-of-the-art docking algorithms showed that ASP score indeed gives higher success rate than the pure shape complementarity score of FTDock but lower success rate than Zdock3.0. We also developed a softly restricting method to add the information of predicted binding sites into our docking algorithm. The ASP-based docking method performed well in CAPRI rounds 18 and 19.

**Conclusions:**

ASP may be more accurate and physical than the pure shape complementarity in describing the feature of protein docking.

## Background

Most proteins interact with other proteins to perform their biological functions in the form of protein complexes. During the past several decades, many docking programs have been developed to predict protein-protein complexes. Among them, the docking algorithms based on Fast Fourier Transform (FFT) are widely used and have made great success[1] because they can search 6D space in a very fast way. These programs include MolFit[2], 3D-Dock[3-5], GRAMM[6], ZDock[7,8], DOT[9], BiGGER[10] and HEX[11]. The base of the original FFT-based docking method is shape complementarity between receptor and ligand. It is usually used as the first step of docking procedure and then other methods are used to refine or re-rank the docked structures [3,12,13]. Besides the FFT-based algorithms, there are other well-known docking algorithms that also consider flexibility of proteins during docking procedure, like RosettaDock[14], ICM-DISC[15], AutoDock[16], or HADDOCK[17]. Since the original FFT docking algorithm only used shape complementarity feature to solve bound docking problem[1], different scoring functions based on other physical features have been integrated into the original FFT-based docking method to improve the prediction ability. For examples, the 3D-DOCK[18] added electrostatic energy into the FFT-based docking method. ZDOCK[7] used atomic contact energy to calculate solvation energy. The hydrophobic docking method [19] combined hydrophobic complementarity with shape complementarity [20]. GRAMM used a long-distance potential[21] to calculate atom-atom van der Waals energy which has proved effective in detecting binding funnels.

Reliable scoring function is crucial to enhance success rate of prediction of protein-protein docking. Cheng and co-workers [22] analyzed the performance of different energy components in protein-protein interactions. They showed that the sum of solvation and electrostatic energies contributes more than 70% to the total binding free energy, while van der Waals energy only contributes less than 10%. Fernandez-Recio's work also showed that rather than electrostatic, van der Waals and hydrogen-bond energies, solvation energy[23] is the most important component in the total binding energy. Zhou et al. [24] found that the correlation coefficient between solvation energy and experimental binding energy is 0.83 with a root mean square deviation (RMSD) of 2.3 kcal/mol, and the most important is that the slope is close to 1 ( slope = 0.93 ).

ASP (Atomic solvation parameters) model is one of the best methods to calculate solvation energy. Due to its fast and efficient feature, ASP model [25-27] has made great success in free energy calculation[28,29], structure prediction[30,31], and scoring functions[22,32]. This suggests that if we integrate ASP into the sampling stage of docking algorithms, it may enhance the success rates of docking. Up to now, several groups have constructed different ASP sets [25-27]. Among them, Zhou's set[24] is the most suitable one for calculating the solvation energy of protein complexes. This ASP set was extracted from 1023 mutation experiments and yielded an accurate prediction of free binding energy of complexes. In this paper, the ASP set from Zhou's work is used to develop an ASP-based protein-protein docking algorithm (ASPDock).

During a prediction procedure, correct auxiliary information (e.g., predicted binding sites) usually can increase the success rate significantly [33-36], but incorrect auxiliary information may mislead predictors and lead to worse predictions. However, we hardly distinguish whether the information is correct or not before the complex structure is experimentally solved. In this work, we present a softly restricting method of using biological information in which we constrain receptor and ligand partially within the predicted binding sites. Using our ASPDock algorithm with softly restricting method, we participated in two rounds of Critical Assessment of PRediction of Interactions (CAPRI)[37]. There are 3 targets (T40, T41, and T42) in rounds 18 and 19. We got high-quality hits for T40 and T41 and the best LRMSD were 2.35 Å and 1.41 Å, respectively.

## Results and Discussion

### Free Energy Calculation

The ASP used here is from Zhou and co-workers [24], which contain only six atom types. It proved to be successful in predicting binding free energy of complexes. ASP model assumes that the solvation energy of an atom or an atom-group is proportional to its solvent accessible surface area (ASA). Accurate calculation of ASA, which depends on the conformation of proteins or complexes, is a time consuming job. In order to meet the speed of the FFT-based docking method, we propose an approximate FFT method to calculate the ASA and so ASP values (see the section "Methods").

We first test our method on the 21 protein complexes[24], whose binding free energies have been measured experimentally. For each complex, we perform a bound docking and select the best structure close to the native state. Usually the LRMSD between the best structure and native structure is less than 0.5Å, and so we consider the ASP score of the best structure as that of the native structure. Using similar method we can calculate the shape complementarity score for each of the 21 complexes. Obviously, if we set all ASP values equal to one, what we calculated in our method is the shape complementarity score.

**Figure 1:**
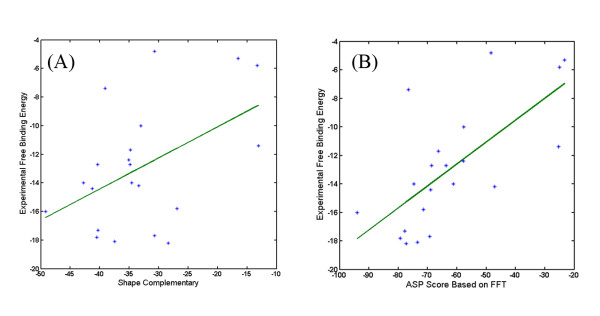
**Correlation between experimental free binding energy and docking score**. (A) Correlation between experimental free binding energy and shape complementarity score. (B) Correlation between experimental free binding energy and ASP score. Calculation of shape complementarity score and ASP score are both based on FFT method. Grid step is 1 Å here.

We compared the ASP and shape complementarity scores with the experimental binding free energy for each of the 21 complexes. The correlation coefficient between the ASP scores and experimental binding energies of the 21 complexes is 0.6868, and that between the shape complementarity scores and experimental binding energies is 0.4843 (Figure 1). In Zhou's work [24], the correlation coefficient between the ASP scores and experimental binding energies of the 21 complexes is 0.83 since they used a more accurate method to calculate the ASA than us. This shows that our approximate method can count most part of the binding free energy and is better than pure shape complementarity method. The later is easily understood because the shape complementarity is a reduced ASP model by taking all atoms as the same.

### Benchmark Calculation

**Figure 2:**
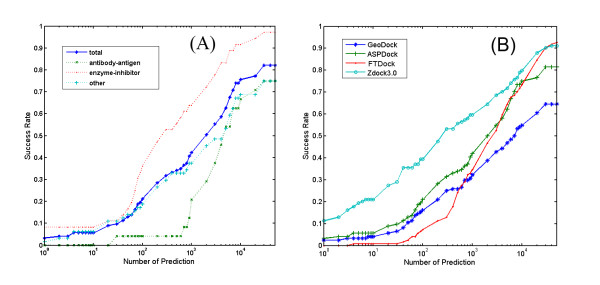
**Success Rate on benchmark 3.0**. (A) Success Rates of ASPDock method on antibody-antigen complexes, enzyme-inhibitor complexes, other complexes and total complexes, respectively. (B) Success Rates of ASPDock, GeoDock (Shape complementarity docking using ASPDock algorithm), FTDock and ZDock3.0 methods on the benchmark 3.0.

Our algorithm is further tested on the benchmark 3.0 [38] by using both the ASP and shape complementarity scores. There are 124 protein-protein complexes, which contain 24 antibody-antigen complexes, 35 enzyme-inhibitor complexes and 65 other complexes. In the docking sampling stage, we use 10 degree as a step for the rotational scanning. Success in top N predictions is defined as that at least one acceptable hit is found in top N predictions. Acceptable hits stand for those predicted complexes with ≤ 10Å LRMSD with respect to the native complex structure. LRMSD is the RMSD between the predicted and native ligand molecules after superposing predicted and native receptor molecules. No predicted and experimental information is used in the docking process. Result shows that ASP method enhances the success rate significantly (Figure 2) in comparison with shape complementarity score.

As in other docking methods, the prediction of enzyme-inhibitor complexes has a higher success rate than antibody-antigen complexes and other complexes. That is mainly because enzyme-inhibitor complexes usually have better interface features than other types of complexes [39]. Success rate of antibody-antigen complexes is not as high as in some other methods [5,7,40]. However, complementarity determining regions (CDR) of antibodies can be predicted by sequence[41]. If we utilize this (CDR) information, success rate of antibody-antigen complexes should be enhanced dramatically. In general, ASP method can enhance the success rate significantly.

We also compared our results with the popular docking algorithms FTDock [5] and ZDock [7,8] using the Benchmark 3.0 (Figure 2B and also Additional file 1). The former can be used to compare the performance of ASPDock relative to a pure shape complementarity method and the later can be used to judge the performance of a single ASP score relative to the best docking method integrating many important factors of protein interactions. The results show that the ASP score indeed gives higher success rate than the pure shape complementarity score of FTDock but lower success rate than ZDock. The former shows that "ASP complementarity" is more reasonable for describing the interface character of protein-protein interaction than pure shape complementarity. The later is expected because ASPDock is only to search a more physical model of pure shape complementarity for protein docking and needs integrating more important factors of protein interactions to get a higher success rate of prediction.

### CAPRI Rounds 18-19

**Figure 3:**
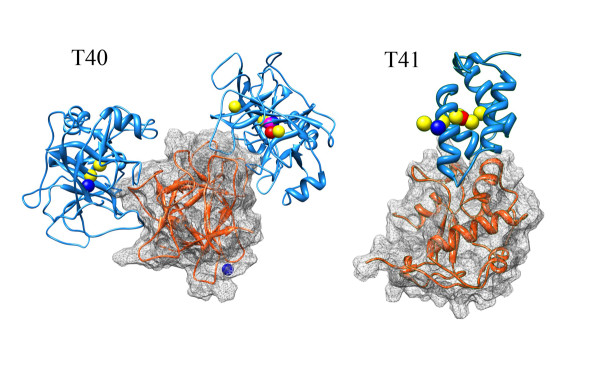
**Native and predicted structures of T40 and T41 in CAPRI**. Our submitted structures are represented by mass center model. Blue balls represent incorrect structures, yellow balls represent acceptable hits, magenta balls represent medium hits and red balls represent high quality hits.

Using our ASPDock and softly restricting method, we participated in the CAPRI rounds18 and 19, which contain three targets, T40 in round 18 and T41, T42 in round 19. We got one high quality prediction for each of T40 and T41 (Figure 3), but no correct prediction for T42. During the docking procedure, we searched the structural space in 5 steps as follows: (1) Searching the binding site information of receptor and ligand from literature; (2) Scanning the six dimensional space by using ASPDock method with the amplified ASP values*ρ*_i_; (3) Picking out the top 2000 structures, clustering them and choosing the structures ranking first in each of the top 20 clusters. In this step, the structures are ranked directly according to their ASP values. (4) Refining the 20 structures using RosettaDock[14] and obtaining a set of new structures; (5) Re-ranking the structures using scoring function, clustering them, and then choosing the structures ranking first in each of the top 10 clusters. The scoring functions we used are RossettaDock[14] and DECK(Shiyong Liu and Ilya Vakser, submitted). The weight of RossettaDock and DECK scores is 1:1.

**Figure 4:**
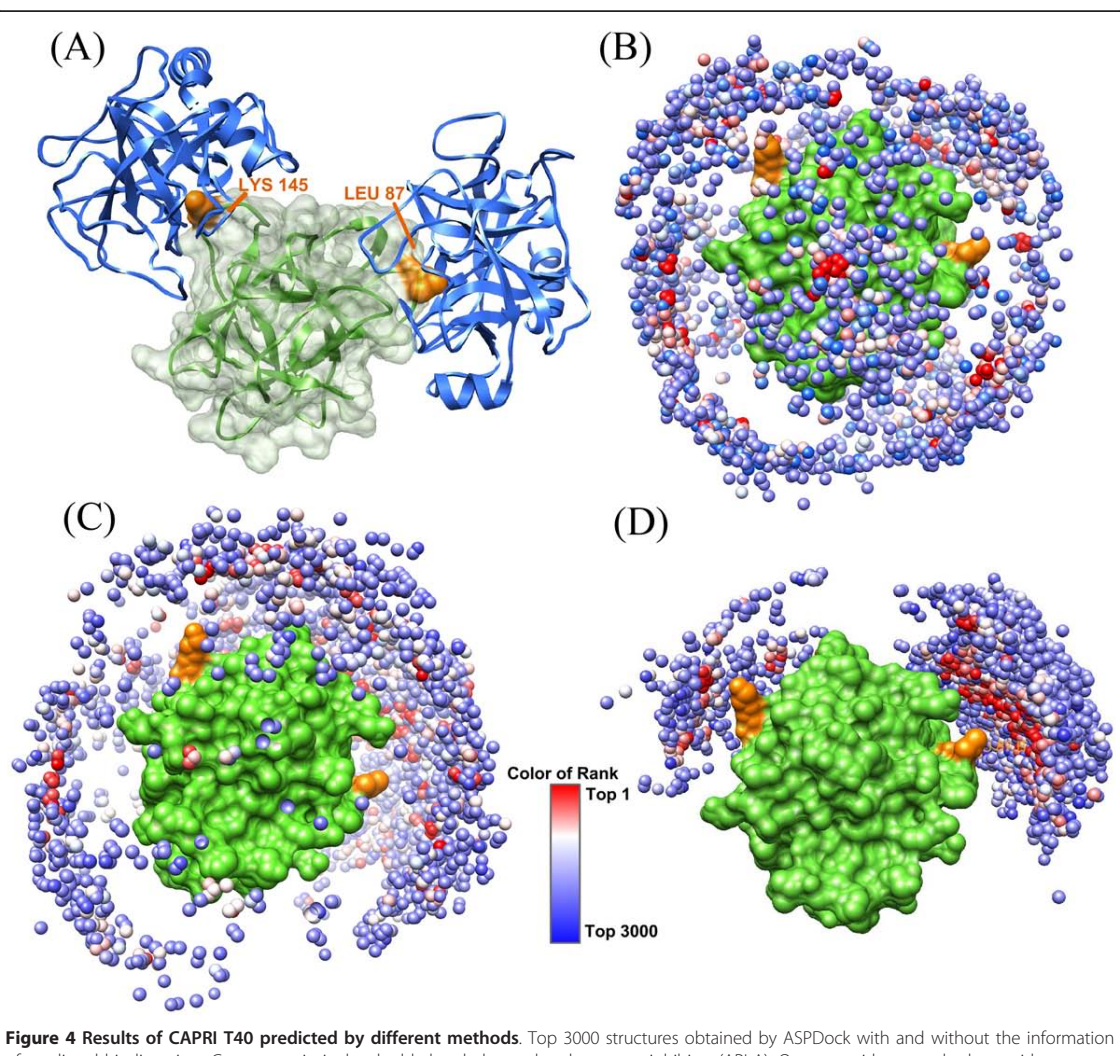
**Results of CAPRI T40 predicted by different methods**. Top 3000 structures obtained by ASPDock with and without the information of predicted binding sites. Green protein is the double-headed arrowhead protease inhibitor (API-A). Orange residues are the key residues, Leu87 and Lys145. Small balls are mass centres of ligands. There are 3000 ligand-mass centres in each figure, representing top 3000 structures of ligands. The ASP scores are ranked form red to blue color. (A) Native structure of T40. (B) Top 3000 ligands generated by shape complementarity method. (C) Top 3000 ligands generated by ASPDock method. (D) Top 3000 ligands generated by softly restricting ASPDock method.

The target T40 (Figure 4) is a complex between bovine trypsin (1BTY) and the double-headed arrowhead protease inhibitor API-A (bound). Some important information shared by Dr. Weng from Boston University shows that the two active sites of the inhibitor are Leu87 and Lys145 (Figure 4A). We incorporated this information into the ASP docking of T40 by using a softly restricting docking method with the amplification factor α being set as 3. For comparison, we also did a totally free docking without using any information of binding sites by shape complementarity method (Figure 4B) and by ASPDock method (Figure 4C). Although free docking can find some structures binding at the residues Leu87 and Lys145, softly restricting ASP docking can greatly enhance the sampling around them (Figure 4D). There is one high quality and six medium hits in our ten submitted structures. The best LRMSD between our hit and experimental measured structures is 2.35 Å.

T41 is the DNase domain of colicin E9 (G95C mutant) in complex with the Im2 immunity protein (C23A/E31C mutant). The unbound coordinates provided are: E9 DNase domain (1FSJ) and Im2 from the NMR ensemble (2NO8). We got one high quality hit and eight acceptable hits in our ten submitted structures. The best LRMSD is 1.41 Å (Figure 3).

T42 is a symmetric homodimer and designed based on Lynn Regan's idealized TPR (1NA3). Residues 1-4 and 108-125 are disordered. We didn't get any acceptable hits of this target (in fact there were only few hits in all predictions from the groups that participated in this CAPRI round).

## Conclusions

We proposed an easy way to incorporate ASP model into FFT protein-protein docking method, which can calculate the solvation energy approximately but quickly. This ASPDock method is reduced the FFT docking method of pure shape complementarity when the ASP values of all atoms are set to be 1. The scores of ASPDock reflect solvation energy, which has proved to be the most significant energy among all kinds of energies in binding free energy. On the contrast, pure shape complementarity has no clear physical meaning. Our results indicate that the ASPDock method can enhance the prediction accuracy significantly in comparison with the pure shape complementarity method.

A softly restricting method was also proposed to incorporate the predicted binding sites into the ASPDock method. This method is more reasonable than the strictly restricting method, which will definitely miss the correct complex structure when the information is incorrect.

## Methods

### Δ*G *Calculation using ASP

Solvation energy of a complex is strongly correlated to its binding free energy. ASP model, first proposed by Eisenberg and McLachlan1[25] in 1986, is one of the most successful models for solvation energy calculation. ASP model assumes that the solvation energy of an atom or an atom-group is proportional to the area of its solvent accessible surface and so the total solvation energy of a molecule is

(1) $$\Delta G=\sum_{i}\sigma_{i}A_{i}$$

where *A*_i _is the solvent-accessible surface area (ASA) of atom *i *and *σ*_*i *_is the ASP value of atom *i*, which can be determined experimentally. Although both analytical [42-45] and numerical [46-48] methods have been developed, accurate calculation of the ASA, which depends on the conformations of proteins or complexes, is still a time consuming job. In this work we propose alternative approach to estimate the ASA quickly and approximately in order to meet the speed of the FFT-based docking method.

**Figure 5:**
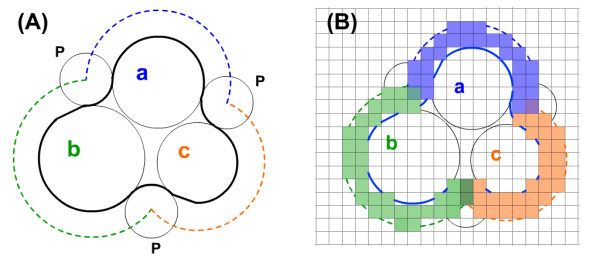
**Calculation of ASP scores using grids**. (A) a, b and c represent different atoms or atom groups, P is the probe molecule, dashed line shows the solvent accessible surface, different colors stand for different ASP values. (B) Projecting these atom groups onto grids, we can calculate accessible surface area approximately by counting grids occupied by the shells of atoms or atom groups.

In FFT-based docking method, proteins are projected onto three dimensional grids (Figure 5). We define the space between molecular surface and accessible surface as a shell. The thickness of this shell is the radius of a solvent molecule, which is 1.7Å for water. We assume that the ASA *A*_i _is proportional to the number *N*_i _of the grids occupied by the shell of atom *i*. If the ASP value of these grids is *σ*_*i*_, we have

(2) $$\Delta G=\sum_{i}\sigma_{i}A_{i}∼\sum_{i}\sigma_{i}N_{i}$$

Obviously $\sum_{i}\sigma_{i}N_{i}$ represents not an absolute but a relative value of Δ*G*, and the accuracy could be controlled by the grid size. Smaller grid size leads to a higher accuracy. Using this approximation, we can quickly evaluate Δ*G *of proteins.

ΔΔ*G ***calculation using ASP**

In order to evaluate stability of a protein complex, we need to calculate the binding energy of complexes ΔΔ*G*,

(3) $$\Delta \Delta G=\Delta G_{\text{Complex}}-(\Delta G_{\text{Receptor}}+\Delta G_{\text{Ligand}})$$

where Δ*G*_Receptor_, Δ*G*_Ligand _and Δ*G*_Complex _are the solvation energy of receptor, ligand and complex, respectively.

**Figure 6:**
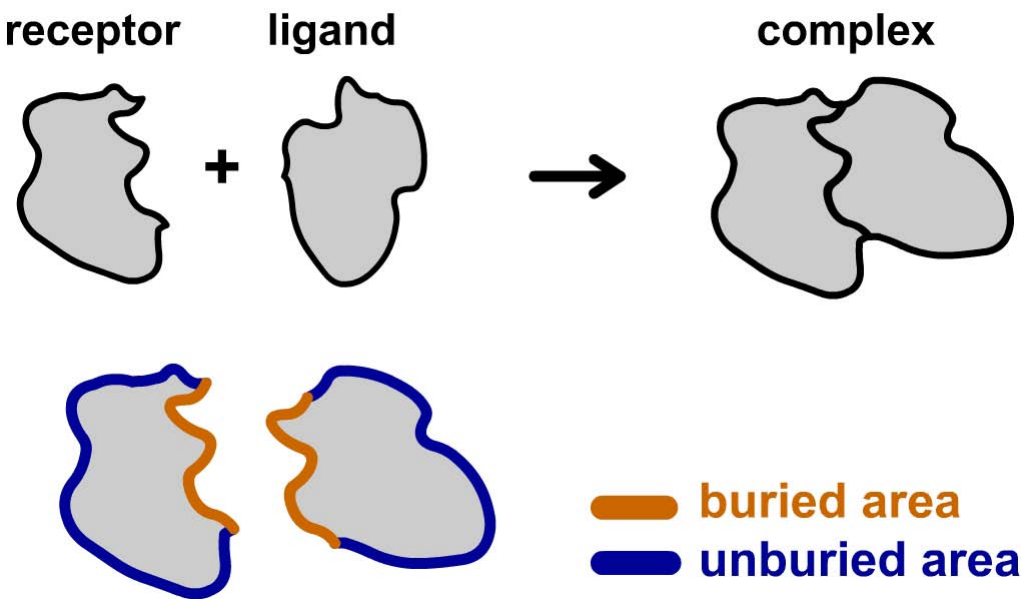
**Schematic of ΔΔ*G *calculation**. Unburied surface keeps unchanged during association in rigid body docking and we can evaluate ΔΔ*G *by only calculating ASP values of the atoms on the buried surface.

For rigid docking problem, the receptor and ligand have no conformational changes during the formation of complex and the calculation of ΔΔ*G *is much simpler (Figure 6). We only need to calculate the ASP values of atoms on the buried surface, i.e.,

(4) $$\begin{array}{l} \Delta \Delta G\text{ = }\Delta G_{\text{Complex}}-\text{(}\Delta G_{\text{Receptor}}\text{+}\Delta G_{\text{Ligand}}\text{)}\\ \text{ = }\sum_{i}^{US}\sigma_{i}A_{i}-\;\sum_{\text{i}}^{TS}\sigma_{i}A_{i}\\ \text{ = }\sum_{i}^{BS}\sigma_{i}A_{i}\\ \;∼\sum_{i}^{BS}\sigma_{i}N_{i} \end{array}$$

Here *TS, BS *and *US *denote the total surface, buried surface and unburied surface, respectively. In this work, we use FFT method to calculate $\sum_{i}^{BS}\sigma_{i}N_{i}$. It is noted that $\sum_{i}^{BS}\sigma_{i}N_{i}$ describes the pure shape complementarity if *σ*_*i *_is set to be 1 for all atoms.

### Fast Fourier Transform

In FFT docking method, receptor and ligand are mapped to three dimensional grids of N × N × N nodes, respectively. The grids occupied by receptor surface, inside receptor, and outside receptor are set as a value of 1, -15, and 0, respectively. The grids occupied by ligand and outside ligand are set as 1 and 0, respectively.

In our ASPDock, receptor and ligand are described by two discrete functions in the same way as follows:

$$\begin{array}{l} R_{ASP}(l,m,n)=\left\{ \begin{array}{l} \text{ ASP value, on the surface}\\ \;\rho +i,\text{ inside of the molecule}\\ \text{ 0, outside of the molecule} \end{array}\right.\\ L_{ASP}(l,m,n)=\left\{ \begin{array}{l} \text{ ASP value, on the surface}\\ \;\rho +i,\text{ inside of the molecule}\\ \text{ 0, outside of the molecule} \end{array}\right. \end{array}$$

where (*l,m,n*) is the grid node coordinates. ASP value used is from Zhou's work[24], which is simple and effective. The value of *ρ *is a penalty for protein overlap, setting from -20 to -40 that doesn't change the results significantly. $i=\sqrt{-1}$ is the imaginary unit.

If Δ*ASP *is defined as the change of ASP values by docking,

(5) $$\Delta ASP=ASP_{receptor}+ASP_{ligand}-ASP_{complex},$$

the score of Δ*ASP *can be calculated using the correlation function:

(6) $$S_{ASP}(\alpha ,\beta ,\gamma )=Im\left[\sum_{l=1}^{N}\sum_{m=1}^{N}\sum_{n=1}^{N}R_{ASP}(l,m,n)\cdot L_{ASP}(l+\alpha ,m+\beta ,n+\gamma )\right],$$

where Im [ ] denotes the imaginary part of a complex function. α, β, γ are the numbers of grid steps by which ligand L is shifted with respect to receptor R in each dimension. *S*_ASP _is positive, zero or negative depending on if there is contact, no contact or overlap between the receptor and ligand after shift. The tolerance of overlap is controlled by the penalty factor *ρ*.

Calculating correlation of two functions directly is a lengthy procedure, which needs *N*^3 ^multiplications and additions for each of the *N*^3 ^possible shifts, and results in totally *N*^6 ^calculations. We use FFT method to accelerate this stage, as in other works.

(7) SASP=Im[1N3IFT(IFT(RASP)·DFT(LASP))]

Here the Discrete Fourier Transform (DFT) and Inverse Fourier Transform (IFT) can be calculated rapidly by using fftw library. Before translational scan, the ligand should be rotated in 360 × 180 × 360 degree. Here we use Lattman's method to delete the redundant angles[5,49], which enhances the efficiency significantly.

After the total scan of transition and rotation, we rank the complexes by their ASP scores in two steps. Firstly, we rank the complexes in each orientation and pick the top N out. Secondly, we rank all the top N complexes together. N could be set from 1 to 10. If N is larger than 10, there will be many similar complexes, which may have no benefit in enhancing success rate of prediction and sometimes even make correct complexes rank worse.

### DECK (Distance and environment-dependent coarse-grained) Scoring Function

Based on Dockground (http://dockground.bioinformatics.ku.edu/), Liu and Vakser developed a low-resolution scoring function for protein-protein docking (Shiyong Liu and Ilya Vakser, submitted). Each residue is represented as one pseudo-atom, the "centroid"of the side chain. The optimal reference state was selected according to the success rate of testing on a public available decoy set (http://dockground.bioinformatics.ku.edu/UNBOUND/decoy/decoy.php).

### Softly Restricting Method

Predicted binding sites can be used to restrict the range of docking sampling and enhance success rate of prediction. This has been done in two ways, using as a post scan filter[3,18,50-52] and integrating into the scanning stage[33,53]. However, the predicted binding sites may be incorrect. This will make the docking prediction completely wrong and much worse than without using the predicted information.

To avoid this problem, we use a softly restricting method to use the predicted information. We amplify the ASP values of the residues belong to the predicted binding sites by a factor α and keep the values of other residues unchanged. The ASP values amplified *ρ*_*i *_are represented as

(8) $$\rho_{\text{i}}=\{\begin{array}{r} \alpha \cdot \sigma_{i},\\ \sigma_{i}, \end{array}\;\begin{array}{l} \text{if atom i belongs to the predicted binding sites}\\ \text{other atoms} \end{array}$$

If α is set as infinity, the docking sampling will be completely restricted to the range of the predicted binding sites. However, if the value of α is finite, the sampling is still allowed around the whole surface of the molecules. Thus, even if the predicted binding sites are wrong, we still have the chance to find the correct docking conformations and the success rate will not decrease significantly.

## Authors' contributions

LL participated in the design of the study, performed computation and analysis, and drafted the manuscript. DG and YH participated in prediction work of CAPRI rounds 18-19. SL helped to improve the programs and draft the manuscript. YX designed and finally wrote the manuscript. All authors read and approved the final manuscript.

## Supplementary Material

Additional file 1 **Docking performance of different programs**. The additional file 1 is a table that contains the detailed data of Figure 4B, i.e., the success rates of ASPDock, GeoDock (Shape complementarity docking using ASPDock algorithm), FTDock and ZDock3.0 methods on the benchmark 3.0. Click here for file
